# Pharmacists’ perceptions of the use of internet-based medication information by patients: A cross-sectional survey

**DOI:** 10.1371/journal.pone.0256031

**Published:** 2021-08-13

**Authors:** Eman Alefishat, Rana Abu Farha, Mohammed Zawiah

**Affiliations:** 1 Department of Pharmacology, College of Medicine and Health Science, Khalifa University of Science and Technology, Abu Dhabi, United Arab Emirates; 2 Center for Biotechnology, Khalifa University of Science and Technology, Abu Dhabi, United Arab Emirates; 3 Faculty of Pharmacy, Department of Biopharmaceutics and Clinical Pharmacy, The University of Jordan, Amman, Jordan; 4 Faculty of Pharmacy, Department of Clinical Pharmacy and Therapeutics, Applied Science Private University, Amman, Jordan; 5 Department of Clinical Pharmacy, School of Pharmaceutical Science, University Sains Malaysia, Penang, Malaysia; 6 Department of Pharmacy Practice, College of Clinical Pharmacy, University of Al-Hodeida, Al Hodeida, Yemen; University of South Carolina College of Pharmacy, UNITED STATES

## Abstract

**Purpose:**

The credibility and the reliability of Internet webpages to seek medication-related information is questionable. The main objective of the current study was to evaluate perception and experience of pharmacists with the use of Internet-based medication information by their patients.

**Methods:**

This is a cross-sectional descriptive study that was conducted to evaluate perception and experience of pharmacists with the use of Internet-based medication information by their patients. During the study period, 200 pharmacists were approached to participate in the study using a paper-based survey to assess their perceptions and current experience with the use of Internet-based medication information by their patients. Data were analyzed using descriptive statistics (mean/standard deviation for continuous variables, and frequency/percentages for qualitative variables). Also, simple linear regression was utilized to screen factors affecting pharmacists’ perception scores of the use of Internet-based medication information.

**Results:**

Among 161 recruited pharmacists, the majority (n = 129, 80.1%) reported receiving inquiries from patients about Internet-based medication information within the last year. Among them, only 22.6% (n = 29) of pharmacists believed that Internet-based medication information is somewhat or very accurate. Unfortunately, only 24.2% (n = 31) of them stated that they always had enough time for their patient to discuss their Internet-based medication information.

Regarding pharmacists’ perception of the use of Internet-based medication information by their patients, more than half of the pharmacists (>50%) believe that Internet-based medication information could increase the patient’s role in taking responsibility. On the other hand, 54.7% (n = 88) of the pharmacists believed that Internet-based medication information would contribute to rising the healthcare cost by obtaining unnecessary medications by patients. Finally, pharmacists’ educational level was found to significantly affect their perception scores toward patient use of Internet-based medication information where those with higher educational level showed lower perception score (r = -0.200, P-value = 0.011).

**Conclusion:**

Although pharmacists felt that usage of Internet-based data by patients is beneficial, they also have believed that it has a negative impact in terms of rising the healthcare cost, and it promotes unnecessary fear or concern about medications. We suggest that pharmacists be trained on principles of critical appraisal to become professional in retrieval information on the Internet that might improve their delivery of healthcare information and their recommendations to patients.

## 1. Background

The Internet websites are one of the leading sources of health education and counseling, and are often used by patients to obtain medical and medication-related information about a particular disease or drug [1–4]. Internet web pages are rapidly growing, and this has been linked with the existence of huge amounts of health information available on these sites, where anyone can access these websites or can build a website to contribute to their available content [3,5].

Nowadays, the dependence on the Internet for health education has increased dramatically, with more than 70% of the United States adult population now using the Internet as the first source for medication-related information [6]. This led to the emergence of the so-called e-patient, where the Internet served as a source to obtain information that the patient failed to obtain from his/her healthcare providers during their clinic appointment [7,8]. From these websites, patients may have access to a large amount of medication-related information which are available freely to them [2].

Care must be taken while searching these websites for medication-related information, especially as many rely on the Internet to seek treatment without resorting to healthcare providers [7,8]. Many web users are turning to the Internet for help with health problems they complain about, but they need to be cautious while doing that, as some Internet sites may be unreliable, and does not provide correct information [8,9]. Also, many patients have a confirmation bias, where they have their own perceptions about their illnesses, and most of the time they are believing information that supports their perceptions, even if they are completely wrong [10].

The increase in the return to the Internet to seek medication-related information was found to negatively affecting healthcare providers-patients’ relationships [11,12]. Pharmacists as healthcare providers lost their traditional role a primary source for medication-related information and they are no longer acting as a safety guards for such information [13]. Also, the credibility and the reliability of Internet-based medication information is questionable [9]. This necessitates pharmacists to take a more dynamic role to guide patients to search, interpret and verify the accuracy and reliability of searched information.

In Jordan, according to the latest statistics by the Jordanian pharmacists Association (JPA), there are 25,700 pharmacists registered in JPA [14,15]. The responsibilities of most of the pharmacists remain focused on dispensing medications, inventory control, and executing physician orders [16]. Besides their responsibilities, pharmacists are still the most competent and qualified healthcare professionals in providing medication-related information [17]. They can support the quality of this role by having a good experience to counsel pharmacy customers about the information they obtain from the Internet and evaluating the quality of the information [18,19].

Information regarding pharmacists’ experiences and perceptions towards the use of Internet-based medication information by pharmacy customers is rather limited in the literature [18,20]. Ong et al. revealed that despite the finding that Malaysian pharmacists showed a positive perception towards patients’ use of Internet-basedhealth information, they were worried about the reliability of the health information from the Internet [20]. In addition, Australian pharmacists realized that they need the appropriate skills to interpret Internet-based health information in a timely efficient manner to improve the overall care provided to the patients [18].

To date, no information is available about Jordanian pharmacists’ experiences and perceptions towards the use of the Internet as a source of medication-related information by pharmacy customers. Thus, the main objective of the current study was to evaluate the perception and experience of pharmacists with the use of Internet-based medication information by their patients, we also evaluated their expected future response to patients’ inquiries about Internet-based medication information.

## 2. Methods

### 2.1 Clinical settings, study design, and subjects

Between May-August 2017, a cross-sectional descriptive study was conducted on community and hospital pharmacists in Amman-Jordan. During the study period, a convenience sample of 200 pharmacists was approached to participate in the study via filling a paper-based survey. Pharmacists were approached at their working sites (including community and hospital pharmacies), and they were informed about the purpose of the study, and that their participation is voluntary, and their response will be kept anonymous. Pharmacists who agreed to participate in the study were asked to sign a written informed consent form.

### 2.2 Sample size calculation

The standard formula: n = P × (1- P) × z^2^/d^2^ was used to calculate minimal sample size where 96 pharmacists were considered a representative sample size for this study. In the absence of literature reporting the prevalence of pharmacists experiencing patients with medical information-seeking behavior, the most conservative proportion 50% prevalence (P) was used, 10% desired precision, and a confidence level of 95% were used.

### 2.3 Ethical consideration

The World Medical Association Declaration of Helsinki guidance was followed in the study [21]. The study was granted ethical approval from the Institutional Review Board (IRB) at the Applied Science Private University (Reference number: 2019-PHA-7).

### 2.4 Questionnaire development

Since no previous studies specifically evaluated pharmacists’ experiences, perceptions and responses to patients’ Internet usage to obtain medication-related information, previous surveys evaluating physicians’ perceptions of their patients’ Internet use as a source of medical information were used to formulate the questionnaire in this study [22,23]. The draft questionnaire was distributed to five pharmacists for content and face validity. Pharmacists’ feedback guided several amendments to the wording of questions to enhance their clarity and a number of statements were modified to ensure the use of a more comprehensive tool.

The final questionnaire contained a total of 32 items (questions or statements) with three major domains of interest including:

- Demographic characteristics (9 questions) including pharmacists’ gender, age, level of education, country and year of graduation, place of work, years of experience, their primary role in the pharmacy, and the number of patients served per day.
- Pharmacists’ experience with patients’ inquiries about Internet-based medication information. Seven close-ended questions were used in this domain, and only pharmacists answered “Yes” to the first question “Did you have experience with patients asking about Internet-based medication information during the last year?, completed the next 6 questions.
- Pharmacists’ perceived attitude to patient use of Internet-based medication information (12 statements), where a Likert scale of 5 (strongly agree (5), agree (4), neural (3), disagree (2), and strongly disagree (1)) was used to evaluate the level of agreement for each positive statement (1–3, 6–8, 11, and 12), while a reverse Likert scale was used for the negative statements (4, 5, 9, and 10) (strongly agree (1), agree (2), neural (3), disagree (4), and strongly disagree (5)). A mean score (out of 5) for pharmacists’ perception was calculated for each pharmacist.
- Pharmacists expected behavior to patients’ inquiries about Internet-based medication information. Four statements were scored out of 5 using the Likert scale described above to evaluate the level of agreement for each statement.

The final questionnaire’s internal consistency was assessed by measuring Cronbach’s α for sections C and D, with values of 0.649 and 0.596, respectively, which indicates acceptable internal consistency.

### 2.5 Statistical analysis

Data were analyzed using the statistical package for social science (SPSS) version 22 (SPSS Inc., Chicago, IL, USA). The mean and standard deviation were used for the descriptive analysis of continuous variables while frequency and percentages were used for qualitative variables. Shapiro-Wilk test was used to test the normality of data (with P-value ≥0.05 indicates a normally distributed continuous variable) [24]. Cronbach’s α was used to evaluate the reliability of the questionnaire i.e. that the scales constructed are fit for their purpose [25].

Simple linear regression was utilized to screen factors affecting pharmacists’ perception scores of the use of Internet-based medication information (with P-value <0.05 indicates significance), and all tests were two-tailed.

## 3. Results

### 3.1 Socio-demographic characteristics of pharmacists

A total of 161 pharmacists agreed to participate, signed the consent form, and filled the questionnaire (response rate = 80.5%). The majority of the study sample were females (n = 115, 71.4%) and had a BPharm or PharmD degree () (n = 137, 85.6%). The mean age of participating pharmacists is 29.7 years, with an average experience of 6.0 years. Most participants were community pharmacists (n = 129, 80.1%) who graduated from Jordan (n = 154, 95.7%). Half of the pharmacists in the study had counseling and dispensing as their main role at the pharmacy (n = 93, 57.9%). A summary of participating pharmacists is provided in Table 1.

**Table 1:** Demographic characteristics of the study sample (n = 161).

Parameters	Mean (SD)	n	%
Age (years)	29.7 (7.4)		
Gender			
• Male		46	28.6%
• Female		115	71.4%
Experience as a pharmacist	6.0 (6.5)		
Educational level			
• BSc (BPharm/PharmD)		137	85.6%
• Graduate studies (MSc/PhD)		23	14.4%
Country of graduation			
• Jordan		154	95.7%
• Others		7	4.3%
Site of work			
• Independent community pharmacy		86	53.5%
• Chain community pharmacy		43	26.7%
• Hospital pharmacy		32	19.9%
Current job responsibilities			
• Managerial		30	18.6%
• Counselling & dispensing		93	57.8%
• Clinical Pharmacy		28	17.4%
• Drug Information		10	6.2%
Number of patients visited pharmacy/day			
• Under 50		66	41.3%
• 50–99		55	34.4%
• 100–149		22	13.8%
• 150–199		8	5.0%
• 200 or above		9	5.6%

### 3.2 Pharmacists experience with patients’ inquiries about Internet-based medication information

**Fig 1:**
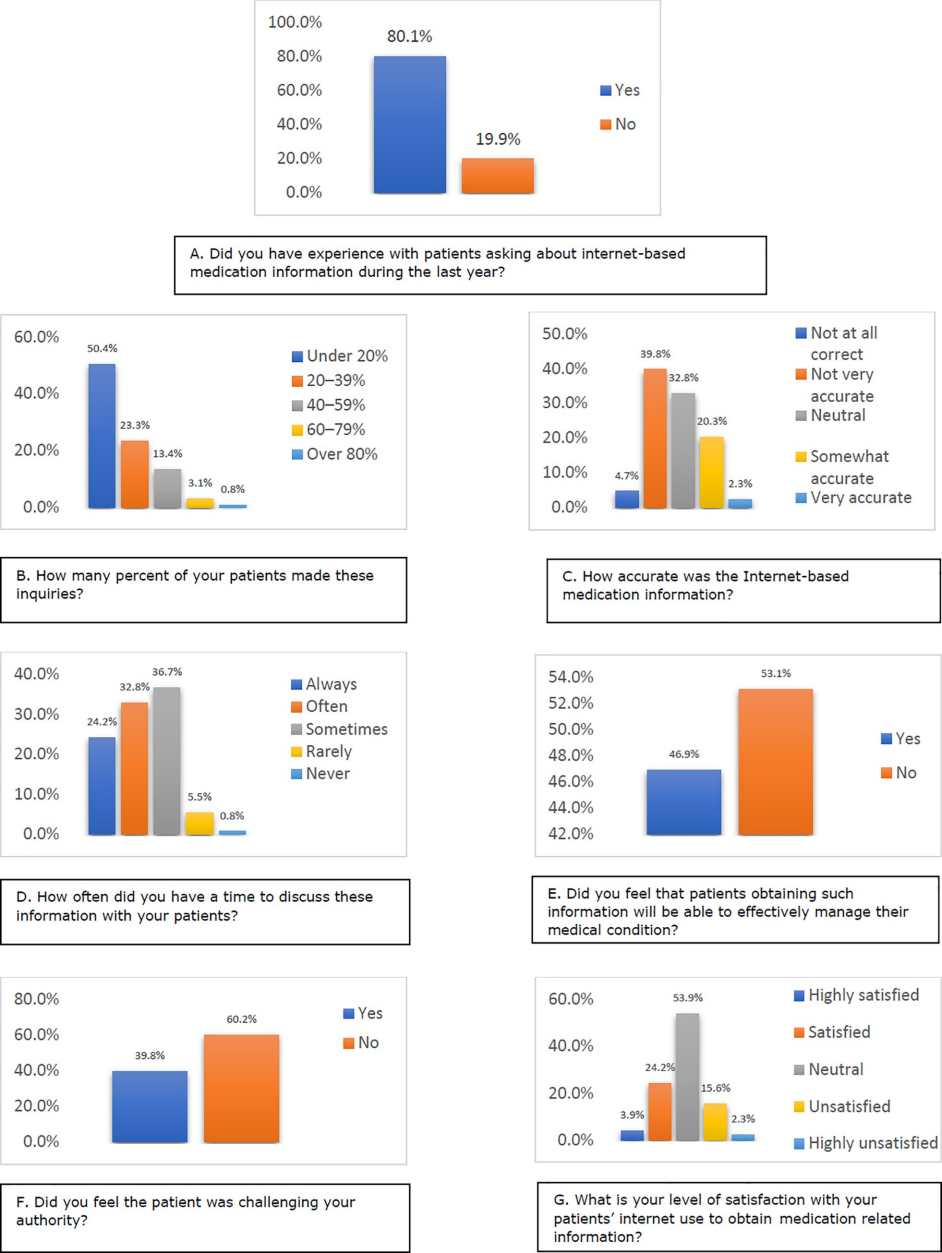
Pharmacists experience with patients’ inquiries about Internet-based medication information, patients who answered the question A by yes (n = 128, 80.1%) were allowed to answer questions from B to G.

A presentation of pharmacists’ experience with patients’ inquiries about Internet-based medication information is displayed in Fig 1. The majority of pharmacists (n = 129, 80.1%) reported inquiries about Internet-based medication information by their patients within the last year. Despite the high percentage of who pharmacists experienced such inquiries, these behaviors are not widely spread, where 73.7% (n = 95/129) of pharmacists stated that less than 40% of their patients had made such inquiries.

Among pharmacists who received inquiries about Internet-based medication information, only 29 (22.5%) believed that the information obtained by their patients is somewhat or very accurate. Unfortunately, only 24.2% (n = 31) of them stated that they always found time for their patients to discuss the Internet-based medication information, while others reported that they often (n = 42, 32.8%), sometimes (n = 47, 36.7%), rarely (n = 7, 5.5%) and never (n = 1, 0.8%) have a time to discuss such information. According to pharmacists’ opinions, only 46.9% (n = 60) of them believed that obtaining such information will enable their patients to effectively manage their medical condition.

Around 40% (n = 51) of pharmacists feel that patients inquiries are challenging their authorities. On the other hand, only 28.1% (n = 36) were satisfied/highly satisfied with the situation when patients present at the consultation with Internet-based medication information.

### 3.3 Pharmacists perceived attitude to patient use of Internet-based medication information

Pharmacists’ perception of the use of Internet-based medication information by patients was grouped into 8 positive and 4 negative statements (Table 2). More than half of the pharmacists (>50%) felt positively toward the usage of Internet-based medication information by patients, where they agreed/strongly agreed that it has positive effects on patients’ confidence during their communication with pharmacists, patients’ role in taking responsibility, patients adherence to medications instructions, patients compliance to treatments of currently under-treated conditions. Statement 8 “The Internet-based medication information causes patients to take up less of their pharmacist’s time” was the only positive statement that has less than 50% level of agreement.

**Table 2:** Pharmacists perceived attitude to patient use of Internet-based medication information.

No.	Statement	Pharmacists’ responses n (%)				
		Strongly agree	Agree	Neutral	Disagree	Strongly disagree
1	The Internet medication information has positive effects on patient’s sense of confidence and control during their interactions with pharmacists	21 (13.1)	73 (45.6)	43 (26.9)	19 (11.9)	4 (2.5)
2	The Internet medication information is proved to increase patient’s role in taking responsibility	17 (10.6)	84 (52.2)	41 (25.5)	18 (11.2)	1 (0.6)
3	The Internet medication information encourages patients to follow medications instructions	21 (13.0)	73 (45.3)	46 (26.6)	20 (12.4)	1 (0.6)
4	The Internet medication information would contribute to rising the healthcare cost by obtaining unnecessary medications by patients	27 (16.8)	61 (37.9)	46 (28.6)	26 (16.1)	1 (0.6)
5	The Internet medication information promotes unnecessary fear or concern about the medications	42 (26.1)	69 (42.9)	37 (23.0)	13 (8.1)	0 (0.0)
6	The Internet medication information encourages patients to have more treatments of currently under-treated conditions	23 (14.3)	80 (49.7)	37 (23.0)	20 (12.4)	1 (0.6)
7	The Internet medication information improves people’s understanding of treatment	19 (11.9)	82 (51.3)	45 (28.1)	12 (7.5)	2 (1.3)
8	The Internet medication information causes patients to take up less of their pharmacist’s time	10 (6.2)	53 (32.9)	35 (21.7)	52 (32.3)	11 (6.8)
9	The Internet medication information causes patients to take up more of their pharmacist’s time	29 (18.0)	62 (38.5)	47 (29.2)	21 (13.0)	2 (1.2)
10	The Internet medication information would damage the good pharmacists–patient relationship	22 (13.7)	37 (23.0)	57 (35.4)	38 (23.6)	7 (4.3)
11	The Internet medication information is accurate in general	7 (4.3)	26 (16.1)	72 (44.7)	45 (28.0)	11 (6.8)
12	Most patients are able to judge the relevance and accuracy of Internet medication information for their conditions	4 (2.5)	29 (18.0)	44 (27.3)	62 (38.5)	22 (13.7)

On the other hand, 54.7% (n = 88) of the pharmacists believed that Internet-based medication information would contribute to the increase in healthcare cost by seeking sometimes unnecessary medications by patients. In addition, 69.0% (n = 111) agreed that Internet-based medication information promotes unnecessary concerns about medications, results in considerable time and effort wasted (n = 91, 56.5%), and it can negatively affect the good pharmacists–patient relationship (n = 59, 36.7%). Also, only 20.4% of the pharmacists thought that the obtained information is generally accurate (n = 33).

### 3.4 Pharmacists expected behavior responses to patients’ inquiries about internet-based medication information

Pharmacists were asked to specify their expected behavior to patients’ inquiries about Internet-based medication information (Table 3), where 86.9% (n = 140) agreed/strongly agreed that they will verify the data and get back to the patient with an answer, 88.2%(n = 142) will check the medical literature or access the Internet to verify the data, 69.6% (n = 112) will use the Internet during patient visits to the pharmacy to look for data to deal with patient problems, and only 23.1% (n = 37) will be annoyed when patients, bring data they found on the Internet.

**Table 3:** Pharmacists expected behavior to patients’ inquiries about Internet-based medication information.

No.	Statement	Pharmacists’ responses n (%)				
		Strongly agree	Agree	Neutral	Disagree	Strongly disagree
	In a situation in which a patient or a family member presents with data they found on the Internet, please categorize your level of agreement					
1	I will promise to verify the data and get back to the patient with an answer	49 (30.4)	91 (56.5)	18 (11.2)	3 (1.9)	0 (0.0)
2	I will check the medical literature or access the Internet to verify the data	49 (30.4)	93 (57.8)	15 (9.3)	4 (2.5)	0 (0.0)
3	I will use the Internet during patient visits to pharmacy to look for data to deal with patient problems	31 (19.3)	81 (50.3)	31 (19.4)	13 (8.1)	4 (2.5)
4	I will be annoyed when patients bring data they found on the Internet	8 (5.0)	29 (18.1)	50 (31.3)	59 (36.9)	14 (8.8)

### 3.5 Socio-demographic factors affecting pharmacists’ perception scores toward patient use of internet-based medication information

A simple linear regression analysis revealed that among the studied socio-demographic factors, only pharmacists’ educational level was found to significantly affect their perception scores toward patient use of Internet-based medication information where those with higher educational level showed lower perception score (r = -0.200, P-value = 0.011) (Table 4).

**Table 4:** Simple linear regression analysis for factors affecting pharmacists’ perception scores toward patient use of Internet-based medication information.

Variables	Perception score	
	Person correlation coefficient	p-value$
Age	-0.084	0.289
Gender [1: males, 2: females]	-0.055	0.448
Years of experience	-0.038	0.642
Country of graduation [1: Jordan, 2: Others]	-0.028	0.728
Educational level [1: BSc, 2: graduate studies]	-0.200	0.011*
Site of work [1: community pharmacy, 2: hospital pharmacy]	-0.011	0.893

$Simple linear regression analysis

*Significant at 0.05 level.

## 4. Discussion

As the most easily accessible members of the healthcare team, pharmacists have shown the highest rate of sharing information with public in comparison with physicians and nurses [26]. For patients, the Internet is considered the primary health care information source [27]. This study was designed to explore pharmacists’ perception and experience of online-based health information that are acquired by patients and communicated with their pharmacists.

In the current study, pharmacists revealed that they have encountered numerous inquiries about Internet-based medication information by their patients. The main concern was that most of the Internet-based information raised by the patients are not accurate enough and not trustworthy, which is concordant to the findings in other studies conducted on both community pharmacists and physicians [22,28]. Studies warned that the issue of accuracy of online retrieved information may result in less literate citizens being more vulnerable to inaccurate information [29]. Therefore, pharmacists should be sufficiently skilled to carry a quick assessment for the quality of online-based medication information to guide their patients to interpret the quality of Internet-based resources [19,30]. Short critical appraisal courses together with training on how to use online medical resources might improve their ability to communicate health care information and recommendations to both patients and other healthcare providers [19,31].

We also found that participants felt that when patients request to discuss online-based medication information, they tend to challenge their authority, which may disrupt or deteriorate the health care professional-patient relationship and health outcomes [11,12,23]. Besides, the majority of pharmacists reported a lack of time to discuss such information with the patients, and the issue may rest with the pharmacists’ stress and hard work since most of the participants receive from fifty to two hundred patients per day, and they may see other general customers and visitors to the pharmacy [31].

Generally, most pharmacists expressed favorable perceptions toward the use of Internet-based health information by their patients, where they believed it has positive effects on patients’ understanding of treatment protocols and their confidence during their interactions with pharmacists. This is consistent with the findings of a previous study conducted on physicians’ responses to patients’ use of online information during the consultation [23]. Our findings also revealed that pharmacists felt that the use of online-based information gets the patient more involved in the treatment decision. This has been reported to increase the patient’s knowledge and improve the health care professional-patient relationship [32]. Nevertheless, our results are not comparable to those of Kim and Kim [22], who found that most physicians felt that Internet-based medication information used by patients might discourage them from following treatment instructions [22]. We believe that communication is a key issue, pharmacists’ communication with patients to verify such information is essential for this use of online information to result in a positive impact on patients’ involvement in treatment decision and their adherence to it. Future studies are necessary to assess the impact of such a role for pharmacists in verifying online information acquired by patients. In agreement with our results, however, in both studies physicians believed that Internet-based medication information would contribute to increasing the healthcare cost by obtaining unnecessary medications by patients, and to promote unnecessary fear or concern about the medications.

Despite the massive workload pharmacists faced, the majority of them reported that they would search the Internet during patients’ visits to the pharmacy to look for information to deal with patient problems. Some pharmacists elected to postpone the verification of unauthenticated information presented by patients and search online resources in their free time. These are not consistent with what was studied on physicians in a previous study, in which they found that most participants do not search the Internet during patients’ visits due to the justification of a very heavy workload of physicians who may see patients at five minutes intervals [23]. Our data indicate that demographic variables did not affect pharmacists’ perception towards the usage of Internet-based medication information by the patients. However, we found that pharmacists’ educational level is significantly affected their perception score, where those with higher educational levels showed lower perception scores. Similar findings were found in a study conducted in Kuala Lumpur, Malaysia, where pharmacists with higher educational levels showed higher uncertainty about the reliability of online health information [33]. It could be since that pharmacists with higher educational levels are more aware of the lack of accuracy of many of the online t-based resources most used by patients and thus they feel that such information may negatively affect patients’ healthcare processes.

Although, this study is the first of its kind in Jordan, which gives it a great strength point, but it has some limitations. Among the main limitation of this study is that the pharmacists were recruited using convenience sampling technique, which may introduce selection bias, and may limit the generalizability of results to the general population of pharmacists. Also, this study relied on pharmacists’ self-assessment of their perception and expected behavior, which could overestimate their perception and actual practice in the real life.

## 5. Conclusion

The majority of the recruited pharmacists had experiences of patients discussing Internet-based medication information. Although pharmacists felt that usage of Internet-based data by patients is beneficial in terms of improving patient’s sense of confidence and understanding treatment, it is crucial to consider that they have also believed that it has a negative impact in terms of rising the healthcare cost, and it promotes unnecessary fear or concern about the medications. Nevertheless, they felt that the overall of their effect on the pharmacist-patient relationship is neutral. We suggest that pharmacists be trained on principles of critical appraisal in order to become professional in retrieval information on the Internet, as well as providing them with specific online health resources skills that might improve their delivery of healthcare information and their recommendations to patients.

## Supporting information

S1 File The study questionnaire. (DOC)

## References

[1] Beranova E, Sykes C. A systematic review of computer-based softwares for educating patients with coronary heart disease. Patient education and counseling. 2007;66(1):21–8. doi: 10.1016/j.pec.2006.09.006 17084058

[2] Hamzehei R, Ansari M, Rahmatizadeh S, Valizadeh-Haghi S. Websites as a tool for public health education: determining the trustworthiness of health websites on Ebola disease. Online journal of public health informatics. 2018;10(3). doi: 10.5210/ojphi.v10i3.9544 30680054 PMC6335090

[3] Bernhardt JM, Hubley J. Health education and the Internet: the beginning of a revolution. Oxford University Press; 2001.

[4] Korp P. Health on the Internet: implications for health promotion. Health education research. 2005;21(1):78–86. doi: 10.1093/her/cyh043 15994845

[5] Evers KE. eHealth promotion: the use of the Internet for health promotion. American Journal of Health Promotion. 2006;20(4):1–14. doi: 10.4278/0890-1171-20.4.1 16555803

[6] Prestin A, Vieux SN, Chou W-YS. Is online health activity alive and well or flatlining? Findings from 10 years of the Health Information National Trends Survey. Journal of health communication. 2015;20(7):790–8. doi: 10.1080/10810730.2015.1018590 26042588

[7] Christensen JKB. The Emergence and Unfolding of Telemonitoring Practices in Different Healthcare Organizations. Int J Environ Res Public Health. 2018;15(1):61. doi: 10.3390/ijerph15010061 29301384 PMC5800160

[8] Ferguson T, Frydman G. The first generation of e-patients. British Medical Journal Publishing Group; 2004. doi: 10.1136/bmj.328.7449.1148 15142894 PMC411079

[9] Sbaffi L, Rowley J. Trust and credibility in web-based health information: a review and agenda for future research. Journal of medical Internet research. 2017;19(6):e218. doi: 10.2196/jmir.7579 28630033 PMC5495972

[10] Giannelli PC. Confirmation bias. Crim Just. 2007;22:60.

[11] Murray E, Lo B, Pollack L, Donelan K, Catania J, White M, et al. The impact of health information on the internet on the physician-patient relationship: patient perceptions. Archives of internal medicine. 2003;163(14):1727–34. doi: 10.1001/archinte.163.14.1727 12885689

[12] McMullan M. Patients using the Internet to obtain health information: how this affects the patient-health professional relationship. Patient Educ Couns. 2006;63(1–2):24–8. doi: 10.1016/j.pec.2005.10.006 16406474

[13] Iwanowicz SL, Marciniak MW, Zeolla MM. Obtaining and providing health information in the community pharmacy setting. Am J Pharm Educ. 2006;70(3):57–. doi: 10.5688/aj700357 17136178 PMC1636958

[14] Rather IA, Kim B-C, Bajpai VK, Park Y-H. Self-medication and antibiotic resistance: Crisis, current challenges, and prevention. Saudi journal of biological sciences. 2017;24(4):808–12. doi: 10.1016/j.sjbs.2017.01.004 28490950 PMC5415144

[15] Ann Nickerson NJMNR, Lauza S. Drug-Therapy Problems, Inconsistencies and Omissions Identified During a Medication Reconciliation and Seamless Care Service. Healthcare Quarterly. 2005;8(Sp):65–72. doi: 10.12927/hcq..17667 16334075

[16] Gala P, Moshokgo V, Seth B, Ramasuana K, Kazadi E, M’Buse R, et al. Medication Errors and Blood Pressure Control Among Patients Managed for Hypertension in Public Ambulatory Care Clinics in Botswana. Journal of the American Heart Association. 2020;9(2):e013766. doi: 10.1161/JAHA.119.013766 31955639 PMC7033820

[17] Kehrer JP, Eberhart G, Wing M, Horon K. Pharmacy’s role in a modern health continuum. Canadian Pharmacists Journal/Revue des Pharmaciens du Canada. 2013;146(6):321–4. doi: 10.1177/1715163513506370 24228046 PMC3819958

[18] Peterson‐Clark G, Aslani P, Williams KA. Pharmacists’ online information literacy: an assessment of their use of Internet‐based medicines information. Health Information & Libraries Journal. 2010;27(3):208–16. doi: 10.1111/j.1471-1842.2010.00891.x 20712715

[19] Bearman M, Bessell T, Gogler J, McPhee W. Educating Australian pharmacists about the use of online information in community pharmacy practice. International Journal of Pharmacy Practice. 2005;13(2):109–15.

[20] Ong SW, Hassali MA, Saleem F. Community pharmacists’ perceptions towards online health information in Kuala Lumpur, Malaysia. Pharmacy Practice (Granada). 2018;16(2). doi: 10.18549/PharmPract.2018.02.1166 30023025 PMC6041214

[21] World Medical A. World medical association declaration of helsinki: Ethical principles for medical research involving human subjects. JAMA. 2013;310(20):2191–4. doi: 10.1001/jama.2013.281053 24141714

[22] Kim J, Kim S. Physicians’ perception of the effects of internet health information on the doctor–patient relationship. Informatics for Health and Social Care. 2009;34(3):136–48. doi: 10.1080/17538150903102422 19670004

[23] Giveon S, Yaphe J, Hekselman I, Mahamid S, Hermoni D. The e-patient: a survey of Israeli primary care physicians’ responses to patients’ use of online information during the consultation. Isr Med Assoc J. 2009;11(9):537–41. 19960847

[24] Tabachnick BG, Fidell LS. Using Multivariate Statistics (5th Edition): Allyn \& Bacon, Inc.; 2006.

[25] Taber KS. The use of Cronbach’s alpha when developing and reporting research instruments in science education. Research in Science Education. 2018;48(6):1273–96.

[26] Lupianez-Villanueva F, Mayer MA, Torrent J. Opportunities and challenges of Web 2.0 within the health care systems: an empirical exploration. Informatics for health & social care. 2009;34(3):117–26. doi: 10.1080/17538150903102265 19670002

[27] Shcherbakova N, Shepherd M. Community pharmacists, Internet and social media: an empirical investigation. Research in social & administrative pharmacy: RSAP. 2014;10(6):e75–e85. 24388002 10.1016/j.sapharm.2013.11.007

[28] See Wan O, Hassali MA, Saleem F. Community pharmacists’ perspectives of online health-related information: A qualitative insight from Kuala Lumpur, Malaysia. Health information management: journal of the Health Information Management Association of Australia. 2018;47(3):132–9. doi: 10.1177/1833358317697718 28537205

[29] Mo P. The use of internet for health education. Journal of Biosafety & Health Education. 2012.

[30] Pohjanoksa-Mantyla M, Bell JS, Helakorpi S, Narhi U, Pelkonen A, Airaksinen MS. Is the Internet replacing health professionals? A population survey on sources of medicines information among people with mental disorders. Social psychiatry and psychiatric epidemiology. 2011;46(5):373–9. doi: 10.1007/s00127-010-0201-7 20225134

[31] McCaw B, McGlade K, McElnay J. The impact of the internet on the practice of general practitioners and community pharmacists in Northern Ireland. Informatics in primary care. 2007;15(4):231–7. doi: 10.14236/jhi.v15i4.663 18237480

[32] Gerber BS, Eiser AR. The patient physician relationship in the Internet age: future prospects and the research agenda. J Med Internet Res. 2001;3(2):E15. doi: 10.2196/jmir.3.2.e15 11720957 PMC1761896

[33] Ong SW, Hassali MA, Saleem F. Community pharmacists’ perceptions towards online health information in Kuala Lumpur, Malaysia. Pharmacy practice. 2018;16(2):1166. doi: 10.18549/PharmPract.2018.02.1166 30023025 PMC6041214

