# Supplementary material for: Pharmacists’ perceptions of the use of internet-based medication information by patients: A cross-sectional survey
Source: PLoS One. 2021 Aug 13;16(8):e0256031. doi: 10.1371/journal.pone.0256031 (PMC8362936; doi:10.1371/journal.pone.0256031)
Supplement: S1 File — (DOC) [file pone.0256031.s001.doc]

| **Pharmacists’ Perceptions of the Use of Internet-based Medication Information by Patients: a Cross-sectional Survey** | |
| --- | --- |
| **Dear Pharmacists**  **Researchers from different universities are carrying out a project to assess the perceptions of pharmacists regarding their Patients’ use of Internet-based Medication Information. This Survey is developed for this purpose. We would like to confirm that all information provided here will be kept confidential, and will be used only for research purpose. Your participation in completing this survey is highly appreciated.**  **Please put a mark on the statement applied to you.** | |
| **Part 1: General information and demographic data of pharmacists** | |
| **Name of Community/Hospital**  **pharmacy** | ___________________ |
| **Gender** | 🞎 Male  🞎 Female |
| **Age** | _______ years |
| **Country of graduation** | 🞎 Jordan  🞎 Others: Specify ___________ |
| **Experience as a pharmacist** | _______ years |
| **Educational level** | 🞎 BSc (BPharm/PharmD)  🞎 Masters  🞎 PhD |
| **Site of work** | 🞎 Independent community pharmacy  🞎 Chain community pharmacy  🞎 Hospital pharmacy |
| **Current job responsibilities** | 🞎 Managerial  🞎 Counselling & dispensing  🞎 Clinical Pharmacy  🞎 Drug Information |
| **Number of patients you provide a service to them/day** | 🞎 Under 50  🞎 50–99  🞎 100–149  🞎 150–199  🞎 Over 200 |
| **Part 2: Pharmacists experience with patients’ inquiries about internet-based medication information** | |
| **For each of the following questions please select the most appropriate answer.**  **1. Did you have experience with patients asking about internet-based medication information during the last year?**  🞎 Yes  🞎 No  **2. How many percent of your patients made these inquiries?**  🞎 Under 20%  🞎 20–39%  🞎 40–59%  🞎 60–79%  🞎 Over 80%  **3. How accurate was the Internet-based medication information?**  🞎 Not at all correct  🞎 Not very correct  🞎 Neutral  🞎 Somewhat accurate  🞎 Very accurate  **4. How often did you have a time to discuss these information with your patients?**  🞎 Always  🞎 Often  🞎 Sometimes  🞎 Rarely  🞎 Never  **5. Did you feel that patients obtaining such information will be able to effectively manage their medical condition?**  🞎 Yes  🞎 No  **6. Did you feel the patient was challenging your authority?**  🞎 Yes  🞎 No  **7. What is your level of satisfaction with your patients’ internet use to obtain medication related information?**   Highly satisfied   Satisfied   Neutral   Unsatisfied   Highly unsatisfied | |
| **Part 3: Pharmacists perceived attitude to patient use of internet-based medication information** | |
| **From the following statements please select your level of agreement with each sentence**   | No. | Statements | Strongly agree | Agree | Neutral | Disagree | Strongly disagree | | --- | --- | --- | --- | --- | --- | --- | | 1 | The internet medication information has positive effects on patient’s sense of confidence and control during their interactions with pharmacists |  |  |  |  |  | | 2 | The internet medication information is proved to increase patient’s role in taking responsibility |  |  |  |  |  | | 3 | The internet medication information encourages patients to follow medications instructions |  |  |  |  |  | | 4 | The internet medication information would contribute to rising the healthcare cost by obtaining unnecessary medications by patients |  |  |  |  |  | | 5 | The internet medication information promotes unnecessary fear or concern about the medications |  |  |  |  |  | | 6 | The internet medication information encourages patients to have more treatments of currently under-treated conditions |  |  |  |  |  | | 7 | The internet medication information improves people’s understanding of treatment |  |  |  |  |  | | 8 | The internet medication information causes patients to take up less of their pharmacist’s time |  |  |  |  |  | | 9 | The internet medication information causes patients to take up more of their pharmacist’s time |  |  |  |  |  | | 10 | The internet medication information would damage the good pharmacists–patient relationship |  |  |  |  |  | | 11 | The internet medication information is accurate in general |  |  |  |  |  | | 12 | Most patients are able to judge the relevance and accuracy of internet medication information for their conditions |  |  |  |  |  | | **Part 4: Pharmacists expected behavior to patients’ inquiries about Internet-based medication information** | | | | | | | | **In a situation in which a patient or a family member presents with data they found on the internet, please categorize your level of agreement** | | | | | | | | No. | Statements | Strongly agree | Agree | Neutral | Disagree | Strongly disagree | | 1 | I will promise to verify the data and get back to the patient with an answer |  |  |  |  |  | | 2 | I will check the medical literature or access the internet to verify the data |  |  |  |  |  | | 3 | I will use the internet during patient visits to pharmacy to look for data to deal with patient problems |  |  |  |  |  | | 4 | I will be annoyed when patients bring data they found on the internet |  |  |  |  |  | | |
